# Methods to promote equity in health resource allocation in low- and middle-income countries: an overview

**DOI:** 10.1186/s12992-019-0537-z

**Published:** 2020-01-13

**Authors:** James Love-Koh, Susan Griffin, Edward Kataika, Paul Revill, Sibusiso Sibandze, Simon Walker

**Affiliations:** 1 Centre for Health Economics, University of York, York, England; 2 East, Central and Southern Africa Health Community, Arusha, Tanzania

**Keywords:** Health equity, Health inequalities, Resource allocation, Economic evaluation, Benefit incidence analysis

## Abstract

Unfair differences in healthcare access, utilisation, quality or health outcomes exist between and within countries around the world. Improving health equity is a stated objective for many governments and international organizations. We provide an overview of the major tools that have been developed to measure, evaluate and promote health equity, along with the data required to operationalise them.

Methods are organised into four key policy questions facing decision-makers: (i) what is the current level of inequity in health; (ii) does government health expenditure benefit the worst-off; (iii) can government health expenditure more effectively promote equity; and (iv) which interventions provide the best value for money in reducing inequity.

Benefit incidence analysis can be used to estimate the distribution of current public health sector expenditure, with geographical resource allocation formulae and health system reform being the main government policy levers for improving equity. Techniques from the economic evaluation literature, such as extended and distributional cost-effectiveness analysis can be used to identify ‘best buy’ interventions from a health equity perspective. A range of inequality metrics, from gap measures and slope indices to concentration indices and regression analysis, can be applied to these approaches to evaluate changes in equity.

Methods from the economics literature can provide policymakers with a toolkit for addressing multiple aspects of health equity, from outcomes to financial protection, and can be adapted to accommodate data commonly available in low- and middle-income settings.

## Introduction

Improving health equity is a key objective for many governments and international organizations [1]. Despite substantial improvements to health system performance in recent decades [2], empirical analyses of health equity have found that inequalities have persisted between and within countries, many of which were described by Marmot and colleagues in their landmark report on global inequalities in 2008 [3]. Victora and colleagues, in a study of 35 low- and middle-income countries (LMICs) found that coverage of skilled birth attendance was more than twice as high in the richest fifth of societies compared to the poorest fifth on average [4]. Wagstaff and colleagues, meanwhile, analysed 64 LMICs over the period 1990–2011 and found that socioeconomic inequalities in health status increased in over half of the countries [5].

Health inequity can be defined as the differences in healthcare access or utilisation, quality of care or health outcomes that are considered avoidable and unfair, such as those associated with socioeconomic status, ethnicity or geographical region. An extensive literature has explored how these inequities are determined socially through factors including income, education and employment [3, 6]. They can be further caused or compounded by the relative under-utilisation of health services by the worst-off and least healthy, a phenomenon known as the inverse care law [7]. Socioeconomically advantaged groups are often better placed to access healthcare services, can afford higher quality services when they do access care and adopt new effective services earlier than those in disadvantaged groups [8]. This means that without targeted implementation, new interventions and policies can inadvertently increase inequities in the population [9].

In order to assist governments and policymakers in making equitable health resource allocation decisions, a range of quantitative methods can be used to measure and analyse health equity. The objective of this paper is to provide an introductory overview of approaches, discuss their utility and limitations and provide examples of their application.

## Overview

Inequality is usually present whichever way a population is divided. However, inequalities are only considered inequitable if they are unfair and avoidable. For example, higher utilisation in low income groups might be fair when their health needs are also greater. Conversely, part of the difference in life expectancy could be regarded as unfair if it is a function of factors or characteristics outside of individual control, such as ethnicity. Deciding whether differences can be considered inequitable therefore requires value judgements about the sources of inequality and will vary according to ethical, political or cultural principles.

In this overview our focus is on the set of economic techniques that can be used for whatever set of value judgements are used by a decision-maker, and hereafter discuss differences in terms of inequalities rather inequities. For a wider discussion of the issues around value judgements in health equity, see Kawachi et al. [10] and Sen [11]. Frameworks for adjusting inequalities based on fairness judgements are provided by Fleurbaey and Schokkaert [12].

In this paper we review a set of methods that can incorporate equity concerns into health resource allocation decisions. Whilst equity remains a common priority for health systems in LMICs, evidence on the equity impacts of policies is often scarce [13]. The methods are organised according to four broad policy questions that they can be useful in addressing:

(i) What is the current level of health inequality?
(ii) Does government health expenditure benefit the worst-off?
(iii) Can government health expenditure more effectively address inequality?
(iv) Which interventions provide the best value for money in addressing inequality?

Questions (i) and (ii) primarily relate to issues of inequality measurement: identifying existing inequalities in the health system such as unequal access, utilisation or ill-health. The techniques relevant to questions (iii) and (iv) can be broadly referred to as policy tools: approaches that can be used to inform actions that address existing inequalities through the allocation of resources.

## What is the current level of inequality?

Quantitative analyses of health inequality use data on healthcare utilisation and costs and health outcomes. As equity is often defined in terms of fairness between social groups, measuring it requires information on demographic and socioeconomic variables such as area of residence, age, sex, income or education level. Incorporating a greater number of variables allows analysts to investigate more nuanced aspects of inequality that affect specific subgroups in society (e.g. women with low education) [14]. The level of inequality can then be estimated using a wide range of metrics that vary considerably in their sophistication.

### Gap measures

Suppose that we have data on health care utilisation (number of visits to a local health clinic), and household assets of a large sample of randomly selected individuals in a population. A simple way to summarise equity might be to take the average level of utilisation in subgroups defined by the level of household assets (for example, by quintiles from the lowest to the poorest households). From this we can calculate the absolute gap (e.g. an average difference of 1.6 visits between the lowest and highest income groups) or relative gap (e.g. a 180% difference in average visits). However, focusing on the gap between the best and worst off ignores information on the link between socioeconomic status and healthcare use from the middle groups.

### Regression-based measures

**Fig. 1:**
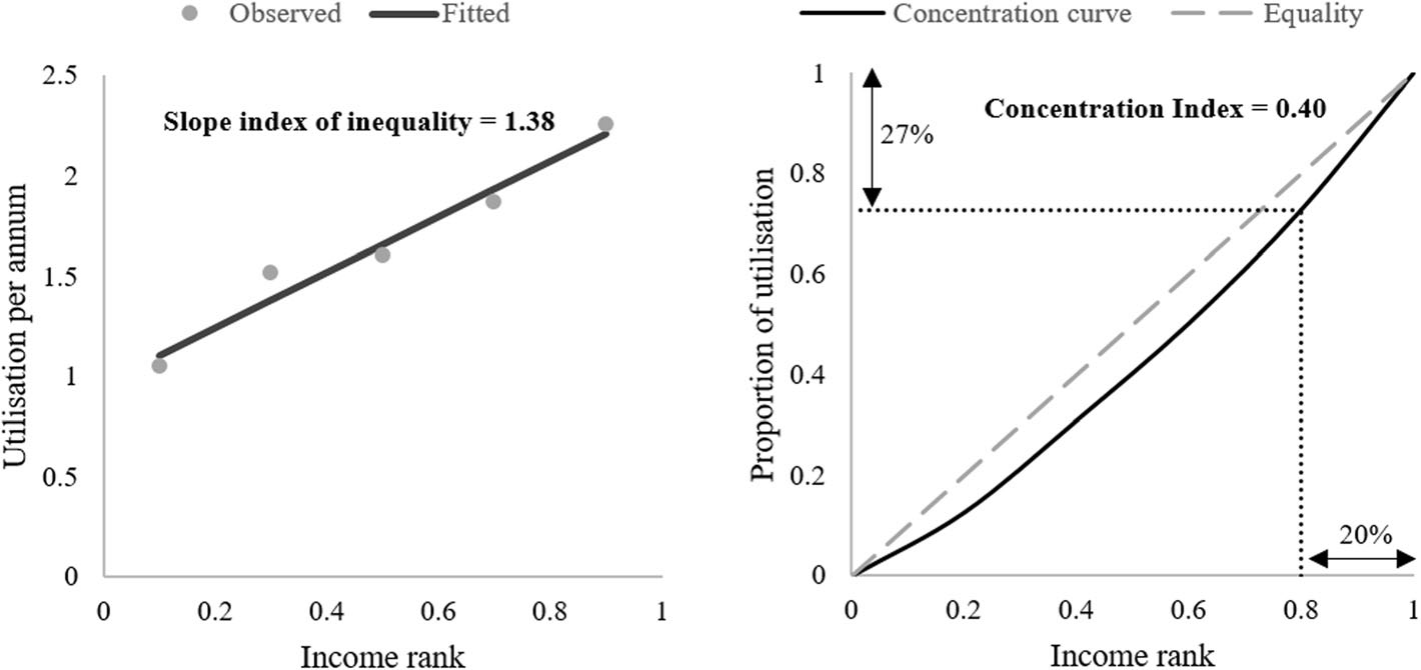
Visualizations of inequality metrics. The left panel shows the line fitted through a cloud of data to generate a slope index of inequality estimate (1.38). The right panel shows the concentration curve of health care utilization. In both instances, low income groups use less health care than high income groups. Notes: 1. The interpretation of the slope index is that expected utilisation increases by 1.38 units as we move from the lowest to highest income group. 2. The concentration index is defined as two times the area between the concentration curve and the line of equality. The former becomes more convex as inequality increases, increasing the area and the concentration index

An alternative approach is to plot healthcare utilisation against household assets, and to fit a line that predicts how utilisation changes with wealth. The slope of this line can be interpreted as the difference in utilisation when moving from the bottom to the top of household asset distribution and is referred to as the slope index of inequality [15]. An example of this is shown in Fig. 1 (left panel). If the relationship between household assets and utilisation is roughly linear, the slope index will be close to the absolute gap. The relative inequality index can be derived from the slope index by dividing through by mean utilisation; this gives the percentage change in utilisation when moving from the bottom to the top of the distribution.

With more than two variables methods such as multivariate regression analysis can be used to estimate the associations between social characteristics and health-related outcomes. These can incorporate multiple equity-relevant characteristics and control variables. The results can be used to ‘decompose’ observed inequality – i.e. to derive the proportion of inequality that is attributable to different characteristics of interest. Charasse-Pouélé and Fournier [78], for example, find that between 57 and 76% of the observed differences in health between ethnic groups in South Africa can be explained by their underlying socioeconomic profile.

### Lorenz and concentration curves

Other metrics are more mathematically complex but can incorporate more information than group averages, providing a richer and more informative measure of inequity. Amongst the most commonly employed of these are the Lorenz curve and the concentration curve. Both these curves illustrate an observed distribution of a health variable against a diagonal line representing a perfectly equal distribution [16]. The distinction between them is the choice of population ranking variables (represented on the x-axis). When the population is ranked according to the level of the health variable itself, a Lorenz curve is produced that displays univariate inequality in health (i.e. cumulative health against health ranking). When the population is ranked according to a different, non-health variable (for example, level of household assets), a concentration curve that summarises bivariate inequality is generated. In the example shown in the right panel of Fig. 1, individuals are ranked in terms of their income. Income rank is then plotted against the cumulative proportion of health care utilisation. For instance, the bottom 20% of the sample account for 11.4% of overall utilisation, whilst the top 20% account for 27%.

The Lorenz and concentration curves can be used to calculate indices that summarise the level of inequality. The Gini coefficient, widely used in the measurement of income inequality, is derived from the Lorenz curve and ranges from 0 (perfect equality – health is shared equally) to 1 (perfect inequality - where one group holds all of the health variable and the remainder have none). The concentration index ranges from − 1 (perfect pro-poor inequality – where the poorest group hold all of the health variable) to 1 (perfect pro-rich inequality – where the richest group hold all of the health variable), with 0 similarly representing perfect equality. The concentration index for the pro-rich distribution in the right panel of Fig. 1 is 0.40. The Wagstaff index [17] and Erreygers index [18] have been proposed as alternatives to the concentration index when the health variable has upper and lower limits (e.g. vaccination coverage rates).

### Measures incorporating inequality aversion

Deriving a single index measure of inequality requires the specification of a set of weights attached to each individual or population group. These weights are then combined with the respective health variable value and summed up to produce the index score. The relative weights attached to individuals in the Gini and concentration indices are (i) embedded in the functional form of the index and (ii) derived from the individual’s rank. The extended concentration index allows for the functional form and the weights to be adjusted by incorporating an additional parameter, denoted *υ*, that defines the degree of inequality aversion [19]. Inequality aversion captures how much we care about reducing inequality: it can be set to a level that it produces the same weights as in the (unextended) standard concentration index (*υ* =2), or such that inequalities do not matter (*υ* =1, where the concentration index is always equal to zero no matter the level of inequality). As *υ* increases above 1, greater weight is applied to those with low ranking, while the proportion of high-ranking individuals with a weight approaching zero increases.

The Atkinson [20] and Kolm [21] inequality indices also incorporate inequality aversion parameters into their formulae. However, both weight individuals in a distribution according to their level of health, rather than their rank in the population. Provided there is some degree of inequality aversion (i.e. that the parameters are greater than zero), improvements to those at the bottom of the distribution are valued more highly. The Atkinson measures the degree of relative inequality, whilst the Kolm measures the absolute difference.

### Health-related social welfare

Several of the inequality measures described above can be combined with average levels of health to provide a measure of health-related social welfare. The general form of these functions is *w* = *μ*(1 − *I*), where *w* is the measure of welfare, *μ* is the mean of the health-related variable and *I* is the inequality measure. A higher value of *I* (greater inequality) will therefore reduce health-related social welfare. The extended concentration index can be used to calculate the health achievement index [22], whilst the Atkinson and Kolm inequality indices are used to calculate their respective welfare indices [20, 21].

By simultaneously incorporating inequality concerns, average gains and inequality impacts, health-related social welfare functions can evaluate policies that include trade-offs between inequality and average health impacts (i.e. where both inequality between groups and average population health increases or where both reduce). The use of these measures requires the specification of the inequality aversion parameter. Whilst experimental methods can be used to elicit values for this parameter [23], sensitivity and threshold analysis can illustrate the critical values for a range of value judgements about inequality.

## Does government health expenditure benefit the worst-off?

### Benefit incidence analysis

Inequalities in the distribution of government health expenditures can be measured using benefit incidence analysis (BIA). ‘Benefits’ in BIA refers not to the improvements in health received by individuals but the financial value of the services they use. For each individual, these are calculated by multiplying the number of times each type of health service is utilised by the respective unit cost. Unit costs are typically estimated from national accounts, with utilisation measured from regional health surveys. User fees, in terms of both insurance premiums and out-of-pocket costs, are then subtracted to calculate the amount of public benefit received.

**Fig. 2:**
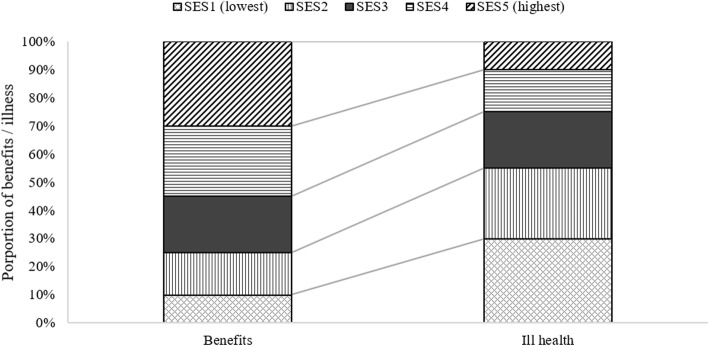
Comparison between the distribution of benefit incidence (left column) and ill health (right column). This shows an inequitable situation in which the lowest socioeconomic groups have the greater health needs but receive lower levels of public health service benefit. **Note**: SES = socioeconomic status

BIA is a technique that has been regularly used by the World Bank and others since the 1990s to answer the question of how public healthcare expenditures are shared between social groups [24–28]. It consists of calculating the share of benefits received by individuals or groups (typically socioeconomic groups) from public health expenditures, as shown in the left column of Fig. 2. Inequalities are then analysed by calculating a concentration index. A step-by-step guide to conducting BIAs can found in McIntyre and Ataguba [29].

The data requirements for conducting a BIA include representative information on utilisation by service type (e.g. primary or secondary care) over a fixed period of time and private expenditures for the population of interest. Including a wider range of services will lead to a more accurate picture of inequality as the analysis can then incorporate more detailed variations in service use; this is, however, governed by the detail provided in national health expenditure accounts, which vary from country to country. Another key assumption is that the cost of a unit of utilisation is independent of the private user fee – in other words, that the service received is identical regardless of how much an individual pays. However, services with user fees attached may be more comprehensive than fully subsidized ones, resulting in higher unit costs. An extension to the standard BIA framework that relaxes this assumption has been proposed by Wagstaff [30].

Evidence on the distribution of public health system benefits does not, however, provide sufficient information to determine how equitable the system is. In order to do this, we also need to know the health care needs of individuals and/or groups. This distribution of need, represented in the right bar in Fig. 2, can be calculated using a variety of metrics. These can be responsive to available data, from simple health survey questionnaire responses on self-assessed health to measures of total disease burden. The latter quantifies all health losses associated with a comprehensive set of diseases [31] and represents the best available standard of evidence on health needs, but does require rich data.

BIAs can be relatively cheap to perform [32], are adaptable to the available data and can provide an informative picture of inequalities over time if they are conducted intermittently. However, they do not provide evidence on whether additional expenditure is likely to improve health inequalities. Increases in expenditure could conceivably go to funding services that are utilised by higher income groups, generating more inequality. In such cases, equity objectives could be pursued by alternative means, such as addressing the social determinants of health inequality or targeting funding toward inequality-improving interventions.

### Marginal benefit incidence analysis

Marginal benefit incidence analysis (MBIA) has been proposed to answer the question of which groups benefit from additional expenditure [33, 34]. MBIA estimates the statistical relationship between benefit incidence (for each social group) and public health expenditures, by comparing the levels of each over time across regions or at a single point in time between regions. As a result, it produces estimates of the expected distribution of benefits that result from increases in expenditure (or alternatively, the ‘losses’ from decreases in expenditure). In an analysis of marginal benefits in Indonesia, Kruse and others find that increases in public health care expenditures benefited the low income groups more substantially than a static analysis would suggest; the bottom quartile received 23% of the benefits in the traditional BIA versus 25% in the MBIA [35]. Conversely the shares of the richest quartile dropped from 24% in the BIA to 21% in the MBIA.

MBIA was developed with a view to examining benefit incidence across the public sector at the margin, although few applications have been made in the health sector. The causes for this low uptake are likely to include greater data requirements and more complicated estimation procedures relative to BIAs. MBIAs require (i) relatively detailed information on public health care expenditures (either over time or across a large number of regions) and (ii) knowledge of appropriate statistical methods to conduct the analysis. The latter is particularly important to avoid biases that can occur when estimating a causal relationship between health behaviours and public expenditure. Reverse causality, for example, is a major concern, as higher health expenditure may be historically allocated in response to poor healthcare outcomes, including utilisation. Not mitigating for these potential biases would therefore produce inaccurate results. A full discussion of these issues can be found in Gravelle and Backhouse [36]; for an applied example that uses instrumental variable regression to address these issues, see Lomas and others [37].

## Can government health expenditure more effectively promote equity?

### Regional funding formulae

A substantial proportion of countries now fund health care using a national public budget. Whether this is funded almost entirely through general taxation or national health insurance scheme or from a combination of public and donor funds, a growing number distribute this budget to decentralised, regional purchasing bodies [38, 39]. The use of mathematical formulae to help allocate the budget originated in the UK in the late 1970s [40] and they are now a feature of health systems in LMICs, particularly in Africa [41–43] and South America [44]. Overviews of the formulae adopted by three African countries is provided in Table 1.

**Table 1:** Overview of three resource allocation formulae in Africa

Country	Year initiated	Description
Malawi	2000	Applies to recurrent, operational health expenditures only. Following a revision in 2008, the budget has been allocated to 28 districts based on a weighted population calculation determined by four factors: outpatient department utilisation, bed capacity, district cost level and the prevalence of stunting (45). The weights attached to each factor are set by health policymakers. Set to be revised in future and will explore ways to align district allocations with the delivery of the Essential Health Package, Malawi’s defined health benefits package (46).
Tanzania	2004	Applied to a pool of donor funds under the ‘Sector Wide Approach’ initiative. Reweights the regional population according to three factors: a mileage index to account for supply costs; under-5 mortality rates as a measure of overall need and the local poverty level to reflect socioeconomic factors. (47). A major part of Tanzanian healthcare funding was reported to stem from ‘block’ grants allocated to regions for multiple public services, and therefore reflected a range of other regional needs besides healthcare.
Zambia	1994	Population-based formula was revised in both 2004 and 2010 to include socioeconomic and geographical factors, respectively (48). Although comparisons between the allocations derived from the formula and actual expenditure have shown large discrepancies, gradual progress toward the ‘equity target’ allocations is being made (49).

One of the central justifications for implementing a resource allocation formula is the promotion of both vertical and horizontal equity: regions with the same health needs are provided with the same resources (horizontal), and regions with different health needs are provided with different resources (vertical). Without formulae, regional budget allocations are based on historical precedent, a phenomenon referred to by Maynard & Ludbrook as “what you got last year, plus an allowance for growth, plus an allowance for scandal” [50]. Furthermore, budgets may also be influenced by favouritism or political importance. By distributing resources in accordance to the health-care needs of the local population, allocation formulae can overcome these issues.

The formulae allocate resources based on the health needs of each geographical area. Many formulae define need in terms of the historical levels of met needs, expressed in terms of health care utilisation [39]. Utilisation for an area is predicted by analysing historical data on health care use. Where utilisation is expected to be higher, a region’s population size will be reweighted to increase their share of resources. An example of this process, from the original English 1976 formula, is shown in Table 2, and a step-by-step guide to estimating and implementing allocation formulae can be found in Mcintyre and Anselmi [38]. It has been recommended that the implementation of formulae allocations should be slowly adjusted from existing levels to the formulae-derived target allocation, so that supply factors can adjust and any potential political ramifications of cutting allocations can be minimised [51].

**Table 2:** Need-weighted populations used in the resource allocation formula for English regions in 1976. A positive difference between the crude and weighted population indicates that health care needs are higher than average and the region requires a greater share of resources

Region	Crude population (000’s)	Weighted population (000’s)	Difference (%)
Northern	3173	3276	3%
Yorkshire	3576	3750	5%
Trent	4661	4594	-1%
East Anglian	1898	1817	−4%
NW Thames	3584	3422	−5%
NE Thames	3874	3757	−3%
SE Thames	3748	3815	2%
SW Thames	2918	3068	5%
Wessex	2816	2773	−2%
Oxford	2403	2118	−12%
South Western	3250	3185	−2%
West Midlands	5342	5153	−4%
Mersey	2543	2655	4%
North Western	4146	4549	10%

Source: Department of Health and Social Security (1976)

Notes: The factors for weighting included age, sex, standardized mortality and hospital bed utilisation

*NW* North west, *NE* North east, *SE* South east, *SW* South west

Formulae can also attempt to account for historically unmet needs that might have prevented individuals from seeking care. Early attempts used relatively unsophisticated area-level mortality statistics, data that are becoming more widely available in LMICs. However, the quality of administrative data should be used with care if there is suspected underreporting. For example, if mortality is underreported in those districts where it is highest, areas with greater needs will not receive adequate allocations. Financial incentives can be utilised to improve data collection at subnational levels, although these should be carefully designed to limit unintended consequences [52].

The proportion of total healthcare expenditure that is financed through tax-funded public resources in LMICs is typically smaller than is seen in high-income countries. For example, analyses in Kenya and Zambia found that the respective resource allocation formulae accounted for 10 and 25% of the total health expenditures only [41, 48], compared with over 60% for the National Health Service in England. The use of ‘basket funds’ that pool donor funding with government budgets are an important tool to increase the equitability of funding across the system. However, pooling frameworks like Sector Wide Approaches [53] can be politically challenging to negotiate, as donors may have a different set of objectives and may wish to allocate resources differently. For example, some donor organisations focus on specific diseases, and allocate their funding to infrastructure and treatments independently of existing health system infrastructure.

Whichever method is used, area-level resource allocation should also attempt to account for differences in healthcare supply between areas, which are likely to be more common and substantial in LMIC settings. This could involve including supply variables (such as mean distance to a hospital or waiting time) in the allocation formula. Gap analyses of regional health system capacity can also be conducted to give an indication of the capital resources required to improve human resources and healthcare facilities to a sufficient level [38].

### Health benefits packages

The increasing popularity of explicitly defined health benefits packages (HBPs) in resource-constrained settings [54] offers an alternative method to traditional formulae when defining area-level allocations. HBPs detail which healthcare services are to be funded from a set of health resources, can therefore provide a way to estimate health resource needs by linking the costs of providing services with the expected target patient population.

Work in Malawi has shown that this can result in substantially different allocations from previous formula-based methods [46]. Disease incidence and prevalence, calculated from household survey data, provided estimates of disease-specific patient populations for each district in Malawi. These were then combined with the costs of providing interventions, which had been previously detailed in an economic analysis of the HBP [55]. Allocation changes of over 50% were recommended for four of Malawi’s 28 districts. Differences in the supply of and access to health services between districts can be explored by comparing an idealistic, fully covered target population and a more realistic, expected level of population coverage.

### Health system reforms

Planners in the health sector may also pursue equity goals through the design of the health system, whether this be through financing mechanisms or the organisation of health services. Typically, the objective of introducing these types of reforms will be to increase access to healthcare or provide financial protection to citizens. These types of activities include introducing community-based or social health insurance schemes [56] or investing in primary care initiatives such as community health worker programmes [57].

Health system reforms, insofar as they have an implementation cost and affect healthcare utilisation and health outcomes, are conceptually akin to highly complex interventions that can be evaluated using the economic evaluation methods outlined in the following section. The most appropriate analysis to undertake will be dependent upon the equity objectives behind the policy [58].

## Which interventions provide the best value for money in reducing inequality?

As well as allocating health resources more equitably, decision makers may similarly care that those resources are spent equitably. This can be achieved by analysing how specific interventions and policies are likely to affect inequalities. A number of tools from the economic evaluation literature can provide quantitative evidence on the comparative impacts of interventions on inequalities and population health.

The prevailing approach to the economic evaluation of health interventions is the use of cost-effectiveness analysis. This framework compares the benefits (expressed in terms of health gain) with the opportunity costs (expressed in terms of the health lost from not funding other interventions) for the average patient. In this section we discuss two prominent alternative approaches that go beyond the average patient to consider impacts across a range of equity-relevant subgroups: extended cost-effectiveness analysis (ECEA) and distributional cost-effectiveness analysis (DCEA). Directly applying weights to health benefits and opportunity costs according to the characteristics of the recipients provides a third alternative [59], although this is yet to be applied in practice and presents a range of practical and methodological issues [60].

**Fig. 3:**
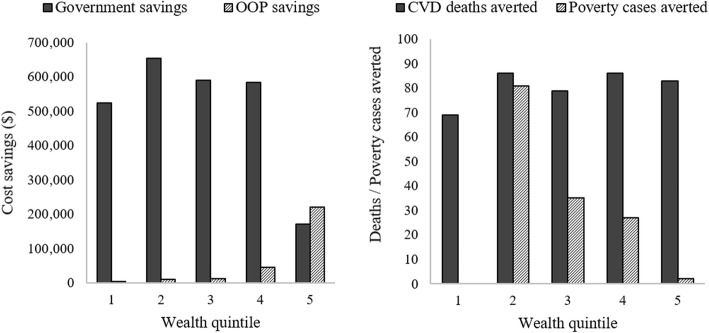
Extended cost-effectiveness results by wealth quintile of a salt reduction policy in South Africa. **Source:** Watkins et al. [62]

DCEA and ECEA both augment the mathematical modelling process of traditional cost-effectiveness analysis to estimate the distribution of impacts of an intervention. Differences in health benefits can be function of differences in underlying health (e.g. morbidities), health-seeking behaviour and expected utilisation. In addition to health outcomes and costs, ECEA also models criteria highly relevant to LMICs settings: financial protection from catastrophic payments and the private expenditures averted from providing the intervention on the public budget [61]. Rather than combine the various criteria, ECEAs present a range of disaggregated outcomes, such as those shown in Fig. 3.

DCEA utilises only two criteria: (i) the total health impact of an intervention and (ii) it effects on health inequalities [63]. Health inequality is modelled prior to an intervention, and the impact of the intervention is modelled to generate a hypothetical ‘post-intervention’ distribution. Although a full DCEA approach uses bespoke estimates of health benefits and costs from a mathematical model, an aggregate, less data intensive approach has also been developed that allocates health benefits to equity-relevant groups based on their share of healthcare utilisation or disease prevalence [64, 65]. Health-related social welfare indices are then applied to evaluate the changes in total health and health inequality in instances where a trade-off occurs (i.e. interventions that improve population health and increase inequality and vice versa).

There are many applications of ECEA in LMICs in Asia, the Middle East and Africa. A wide range of health interventions have been analysed, including neonatal care [66], tuberculosis treatment [67] and rotavirus vaccination [68]. Early applications of DCEA have been limited to high-income countries [69], although an analysis of rotavirus vaccination has also been developed for Ethiopia [70].

## Discussion

Health equity is a complex topic that deals with questions of social value as well as fact [12]. Inequalities related to the health sector can usually be found however one chooses to stratify a population, whether that be by age, sex, income, ethnicity or another characteristic [71]. The methods discussed in this paper can be applied to whatever set of characteristics are relevant to a society and can be used to help inform a range of health resource allocation decisions. BIA can give a broad picture of inequality and can be conducted using commonly available data on healthcare utilisation and expenditure. An MBIA can inform decisions about the impact of changing expenditure but requires additional data and statistical expertise. In terms of evaluating policies and interventions, DCEAs provide the thorough estimation of health inequality impacts, and ECEA can provide information across a range of outcomes – commonly financial risk protection and health. The evidence produced by ECEAs and DCEAs can feed into the deliberative processes used by health policymakers to allocate health resources. Incorporating them into structured processes such as health equity impact assessments [72] is also feasible.

Needs-based resource allocation formulae also form a central policy tool for promoting health equity. As these have been widely implemented (with differing degrees of sophistication) across the world, the principal technical challenge is to collect and utilise better data that will provide more accurate indicators on healthcare needs. The other main challenges are largely political: (i) to bring a greater proportion of total healthcare expenditure under the purview of the formulae through the creation of donor funding pools and (ii) to reduce political pressure and interference. The latter could be achieved by engaging with stakeholders to increase support for the formulae [72] and explicitly setting intermediate annual targets that bridge current and target allocations.

Different combinations of the methods presented can be utilised depending on the type of equity objective that is being pursued. When this is increasing access to healthcare, BIA can be used to assess the extent to which utilisation differs from need, whilst a resource allocation formula can direct resources to where need is greatest. Alternatively, if the objective is to reduce unfair health outcomes, then a resource allocation formula can give extra weight to associated factors such as socioeconomic deprivation [51], whilst DCEAs can identify the interventions that best reduce disparities. ECEA can similarly be utilised to assess impacts on financial protection and poverty; key indicators for systems working towards universal health coverage [73].

The choice of which inequality measures to adopt will depend upon the availability of data and the decision-making context. Simple gap statistics and the slope index of inequality can be simpler to compute and are more easily interpretable by a non-technical audience. However, by assuming a linear relationship between the health outcome and the equity characteristic (slope index) or ignoring data in the middle of the distribution (gaps), these measures can produce misleading estimates of the level of inequality. Many of the methods discussed in this paper, from BIA to DCEA, evaluate inequalities using more technically complex measures such concentration index or health-related social welfare indices.

A critical challenge common to all types of health equity analysis in LMICs is the availability of data. Inconsistent or entirely absent administrative data or vital statistics collection processes, particularly in the poorest countries, represents a major obstacle to conducting robust research. To compound matters, the incentives for improving data collection may be prohibitively costly for the countries’ most in need of them. Regional or country specific health surveys, such as the Demographic and Health Surveys program [74], can also be used to obtain the distribution of a range of health and health service indicators. The World Bank Health Equity and Financial Protection Indicators (HEFPI) dataset, which compiles data from over 40 health surveys, provides a useful starting point for applying many of the methods outlined in this paper.

The incorporation of equity concerns in the design and implementation of HBPs is an emerging consideration in LMICs. A review of 13 packages in Africa noted that, although equity was a key motivation behind the design of the package, evidence-based methods for considering the equity impacts of the included interventions was inconsistent [75]. The economic evaluation approaches we have described can be used to inform the design of future HBPs. Similarly, resource allocation formulae techniques may useful in equitably allocating regional health budgets so that local budget holders hold sufficient resources to provide the local populace with the interventions in the HBP [46].

The focus of this article has been on the effects of health sector decisions on within-country inequalities. Many of the methods have scope, however, to be adapted to investigate between-country differences and resource allocation outside of the health sector. For example, investment decisions by international donor organizations or government departments responsible for overseas aid could evaluate potential investments in terms of how they affected global inequalities in healthcare access and utilisation, whilst policies in education or taxation can be evaluated in terms of their health consequences [76].

Our review does not represent a comprehensive overview of health equity measurement and assessment. We have concentrated on those methods most commonly applied and our literature searches were necessarily pragmatic given the breadth of literature on health equity and resource allocation. We have presented the use of methods in the context of a single, authorised decision-maker, and have not covered their role in more complex decision-making contexts of multiple actors with differing authority and objectives.

## Conclusion

This paper is intended to provide an overview of the methods available to assess and address health equity. We have described the intuitions underlying the approaches and what types of equity questions they address. More than ever, healthcare decision makers can now utilise quantitative analysis to generate evidence to support their pursuit of this important social objective.

## Data Availability

Not applicable.

## Abbreviations

BIA: Benefit incidence analysis
DCEA: Distributional cost-effectiveness analysis
ECEA: Extended cost-effectiveness analysis
HBP: Health benefits package
LMIC: Low- and middle-income country
MBIA: Marginal benefit incidence analysis
